# The Challenges of Ultrasound-guided Thoracic Paravertebral Blocks in Rib Fracture Patients

**DOI:** 10.7759/cureus.7626

**Published:** 2020-04-10

**Authors:** Richa Wardhan, Sowmya Kantamneni

**Affiliations:** 1 Anesthesiology, University of Florida, Gainesville, USA; 2 Anesthesiology, Veterans Affairs Puget Sound Health Care System, University of Washington, Seattle, USA

**Keywords:** ultrasound, rib fractures, nerve block, thoracic paraverterbral block, chest trauma

## Abstract

Thoracic paravertebral blocks (TPVBs) provide an effective pain relief modality in conditions where thoracic epidurals are contraindicated. Historically, TPVBs were placed relying solely on the landmark-based technique, but the availability of ultrasound imaging makes it a valuable and practical tool during the placement of these blocks. TPVBs also provide numerous advantages over thoracic epidurals, namely, minimal hypotension, absence of urinary retention, lack of motor weakness, and remote risk of an epidural hematoma. Utilization of both landmark-based and ultrasound-guided techniques may increase the successful placement of a TPVB. This article reviews relevant sonoanatomy as it pertains to TPVBs. However, certain patient-related issues, including pneumothoraces, surgical emphysema, body habitus, and transverse process fractures, all may make imaging with ultrasound challenging. The changes noted on ultrasound imaging as a result of these issues will be further described in this review.

## Introduction and background

Thoracic paravertebral block (TPVB) is an effective technique for pain management in patients with rib fractures. Even though thoracic epidurals are considered the gold standard for this patient population, the use of low-molecular-weight heparin (LMWH), spine, and other bony fractures preclude the utilization of thoracic epidurals in this group of trauma patients [1]. TPVBs have an important role to play when epidurals are not a good fit.

TPVBs can be placed by landmark-based techniques or with the help of ultrasound (US). Available literature suggests that TPVBs may be performed with a high probability of success using US guidance as an adjunct to traditional techniques [2]. However, there is a paucity of data on the use of real-time US guidance for the TPVBs. In fact, due to a lack of studies, Abrahams et al. gave a grade B level of evidence recommendation for the use of US to place paravertebral blocks based on two small case series. They further stated that there was insufficient evidence to show that US guidance improved block success rates or reduced the risk of complications compared with traditional techniques for performing single-shot or continuous paravertebral blocks [2].

## Review

While US guidance is ideal in the rib fracture patient population to avoid TPVB-associated complications, such as pneumothorax/hemothorax, US-guided TPVBs have their own challenges in this subgroup. Factors that compromise successful placement of US TPVBs include pneumothorax, surgical emphysema from subcutaneous air, morbid obesity, technical issues like an ultrasound device with low resolution, blood or fracture in the intercostal space, and morbidly obese patients [3]. 

To better comprehend these issues, it is important to understand the sonoanatomy of the thoracic paravertebral space (TPVS) and the technical aspect of placing US-guided TPVBs demonstrated herein on healthy volunteers.

US-guided TPVBs can be performed using two approaches: 1) a parasagittal approach and 2) an intercostal approach.

**Figure 1:**
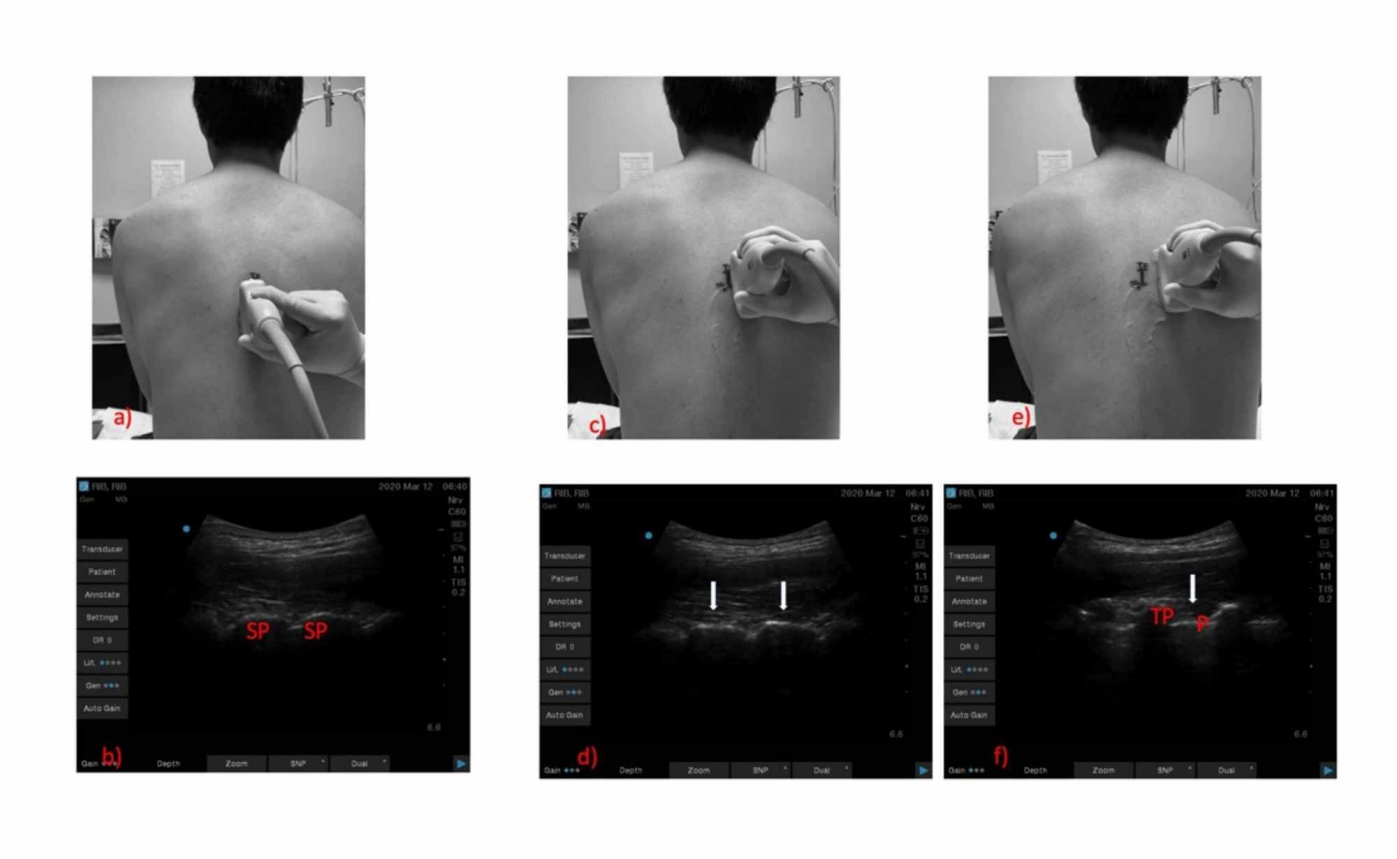
Scanning technique to obtain the parasagittal/longitudinal view on a healthy volunteer a, b) The probe is positioned in the midline longitudinal plane showing spinous processes (SP); c, d) The probe is positioned in the paramedian longitudinal plane depicting the lamina (white arrow); e, f) Moving the probe further lateral shows the transverse processes (TP), pleura (P), and paravertebral space (white arrows)

**Figure 2:**
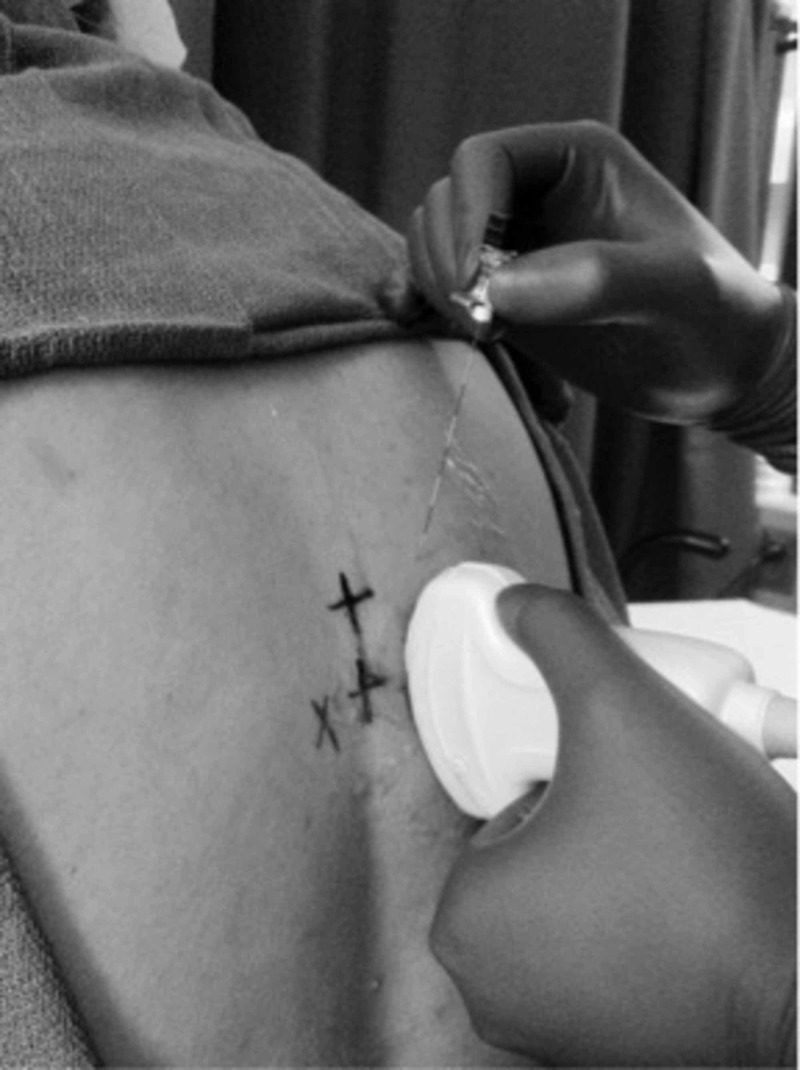
Parasagittal needle approach depicted on a healthy volunteer Paravertebral blocks showing the orientation of a low-frequency probe in the longitudinal plane with the needle approaching cephalad to the probe

**Figure 3:**
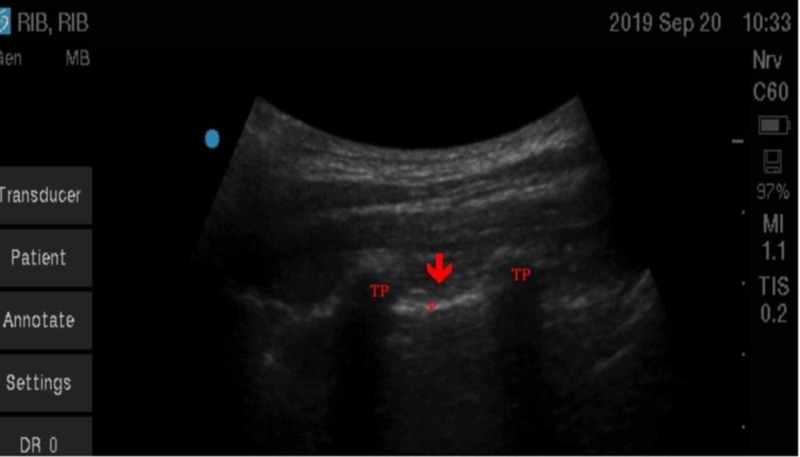
Ultrasound view of paravertebral space (red arrow) in the parasagittal view showing two transverse processes (TP) and pleura (P) in a healthy volunteer.

For the parasagittal approach, a low, curved low-frequency (in obese patients) or a high-frequency linear array ultrasound probe is placed longitudinally, such that the long axis of the probe is parallel to the midline (Figure 1). The position can be sitting or lateral decubitus (Figure 2). The probe is used to scan starting from the midline, identifying the spinous processes and then the lamina, followed by the transverse processes. The pleura appears as an echogenic, shimmering structure in between the transverse processes (Figure 3). The midpoint of the transducer is then aligned midway between the two adjacent transverse processes. Local anesthesia is infiltrated at the superior edge of the probe. Using an in-plane approach, an 18-g Tuohy needle is inserted and advanced between the costotransverse ligament and the pleura (Figure 2). Five mL of ropivacaine 0.5% can be then injected after careful aspiration, and a multi-holed epidural catheter threaded 4 - 5 cm into the space.

**Figure 4:**
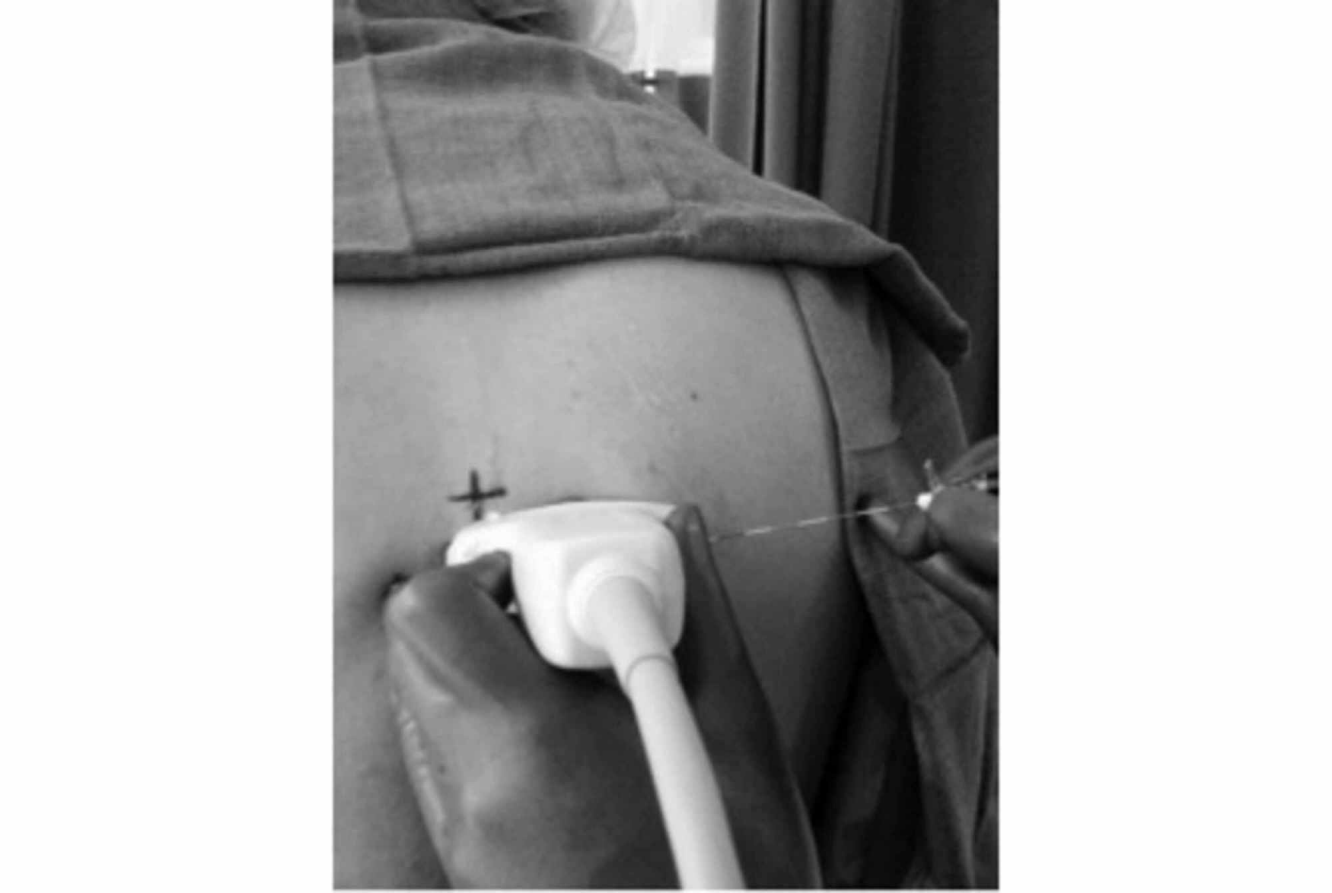
Intercostal placement of the ultrasound for a paravertebral block with the needle approaching lateral to medial shown here on healthy volunteer

**Figure 5:**
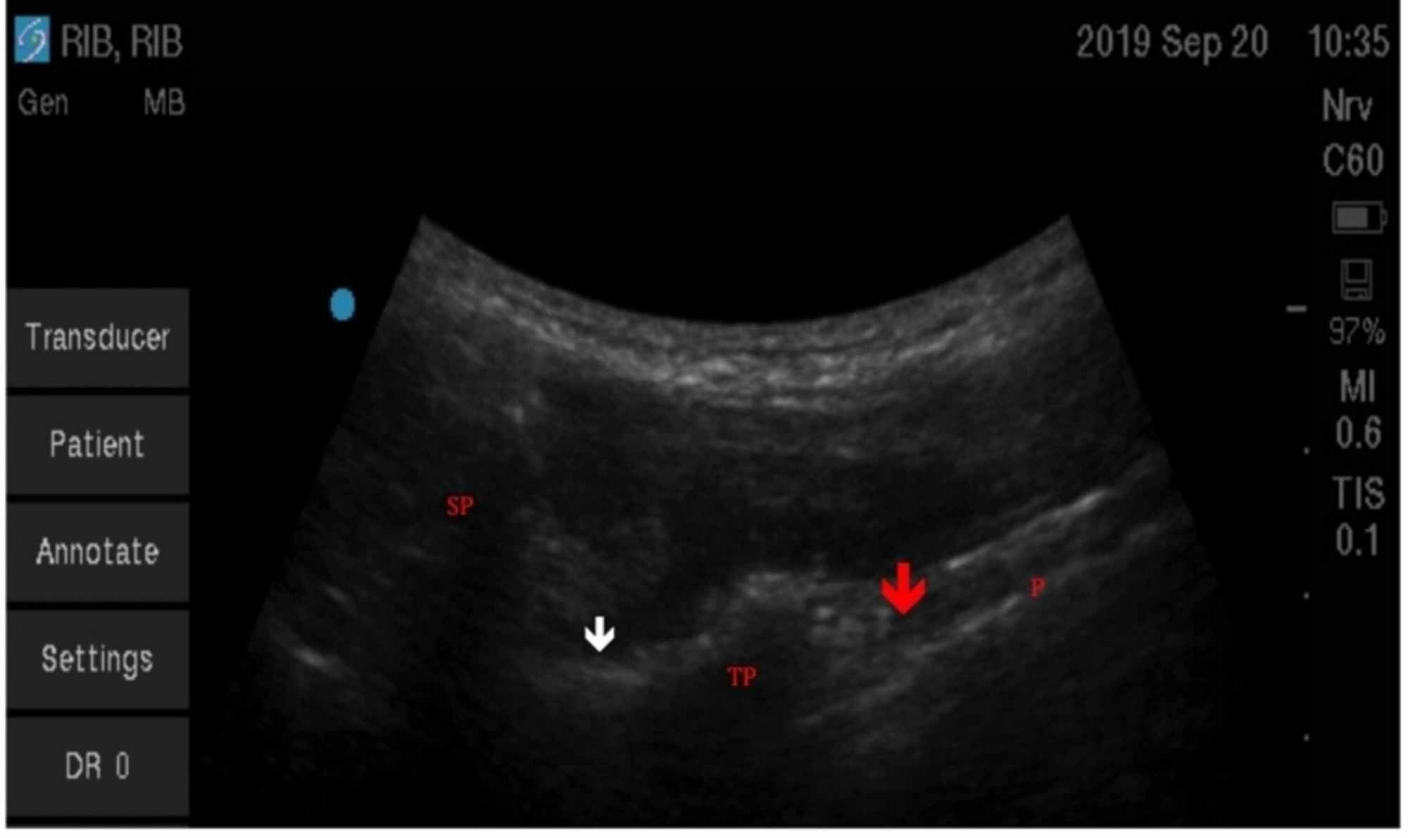
Ultrasound view of the paravertebral space (red arrow) in the intercostal view showing the transverse process (TP) and pleura (P) Spinous process (SP) and lamina (white arrow) are also seen in this view obtained from a healthy volunteer.

For the intercostal approach, we follow the technique described by Shibata et al. [4]. The position can be either sitting or lateral decubitus. A high-frequency linear array probe or curvilinear low-frequency probe (depending upon the body habitus) is placed on the rib at the selected level. The medial edge of the probe is kept close to the spinous process in order to view the transverse process (Figure 4). In this position, the horizontal view of the rib is visualized as a hyperechoic line. The probe is then moved caudally into the intercostal space between adjacent ribs. In this acoustic window, the inferior part of the transverse process can be visualized as a hyperechoic convex line. The apex of the TPVS is visualized as a wedge-shaped hypoechoic space. The TPVS lies enclosed by the hyperechoic line of the pleura below and the internal intercostal membrane above. The apex of TPVS is laterally continuous with the intercostal space (Figure 5). An 18-gauge Tuohy needle is inserted in a lateral-to-medial direction using an in-plane approach and advanced until the needle tip penetrates through the internal intercostal membrane. After a negative aspiration test for blood, 5 mL of local anesthetic is injected into the TPVS slowly and a multihued epidural catheter is threaded 4 - 5 cm in the space. The pleura is seen being pushed ventrally during a local anesthetic injection [4].

Pneumothorax (PNX)

The presence of PNXs in patients with rib fractures is a common occurrence. Ultrasound imaging has been utilized by emergency medicine and critical care physicians to detect PNX for more than two decades now. An ultrasound allows the detection of small amounts of loculated pleural fluid in amounts as small as 20 mL, which cannot be identified by x-rays, as it is only capable of detecting volumes above 50 mL [5]. The knowledge of an ultrasound-guided lung examination is also helpful for a regional anesthesiologist to identify an occult PNX as this could affect the placement of ultrasound-guided paravertebral blocks.

**Figure 6:**
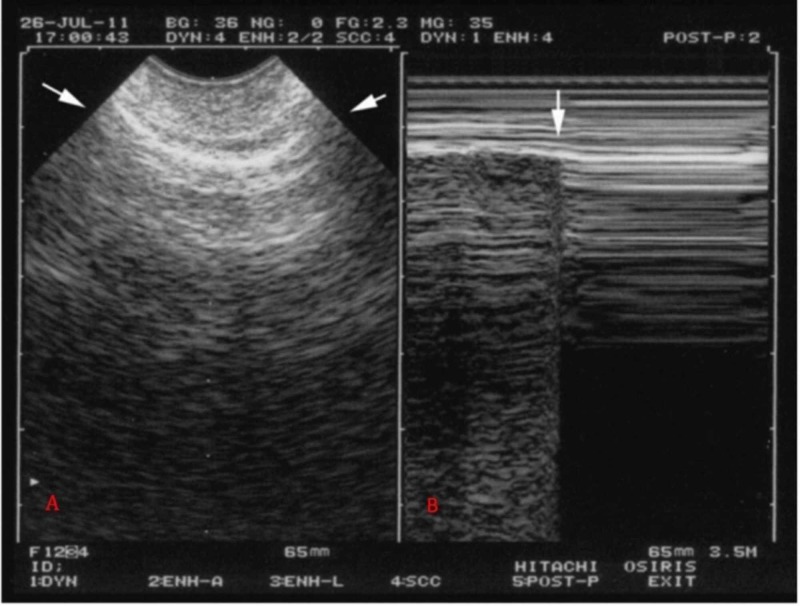
Ultrasound of the third intercostal space along the anterior axillary line The lung point (ultrasound of the 3rd intercostal space along the anterior axillary line). A) Expiration. Absence of lung sliding, plus A-lines; B) M-mode shows the sudden (arrow) inspiratory appearance of the lung point. Permission to use this figure obtained by Lichtenstein et al. [7]

The presence of PNX is determined on the basis of accepted sonographic criteria [6]:

1. Disappearance of lung sliding (Figure 6)

2. Identification of the lung point (Figure 6)

The visualization of lung sliding on the anteroinferior chest wall in the supine patient has a 100% negative predictive value in the diagnosis of PNX [6]. Lung sliding refers to the shimmering appearance of the pleura caused by rubbing of the parietal and visceral pleura on each other on the ultrasound image. When air separates the two pleural layers, the movement disappears and cannot be detected, and the parietal pleura is still visualized but does not move [8]. The presence or absence of the dynamic pleural lung sliding, captured on a single static image, can be further verified using a power color Doppler. The absence of a power color Doppler signal (coined the “power slide”) at the thoraco-pleural interface may serve as a criterion in the identification of a PNX [9].

Horizontal, regularly spaced, hyperechogenic lines representing reverberations of the pleural line are called ultrasound A-lines; narrow-based comet-tail artifacts arising from the pleural line and spreading up to the edge of the screen are ultrasound B-lines. Absent lung sliding, plus exclusive A-lines, are suggestive of pneumothorax.

An absent sliding sign does not always signify that a PNX is present. In critically ill patients, it may just represent the presence of atelectasis. Knowledge of identifying B-lines, also called comet tail artifacts, can be very helpful in such situations. B-lines arise from the pleural line and spread vertically like echogenic rays. They reach the lower edge of the screen without waning and move with the respiratory movements [8]. It may indicate other lung pathology (such as thickened interlobular septa in the interstitial syndromes, like pulmonary edema and lung contusions), but visualization of B-lines rule out PNX with a true negative rate of 100% [8, 10].

The absence of both lung sliding and B-lines in the anteroinferior chest area suggests pneumothorax. Moving the ultrasound to the inferolateral chest wall looking for the “lung point” (which is a point where lung sliding or B-lines are visualized again) confirms the diagnosis of PNX [6].

It is important for the operator performing the ultrasound-guided paravertebral block to recognize a PNX using the criteria above. The presence of lung sliding helps the operator to determine the exact location of the paravertebral space which lies superior to the pleura (extra-pleural) on the ultrasound image. However, the absence of a sliding sign can lead to confusion in identifying the pleura accurately, thus making it difficult to identify the paravertebral space. In this situation, depending upon the amount of air, the probe can then be moved to explore an intertransverse process window cephalad or caudad to place the block where pleura seems more identifiable. 

Surgical emphysema

Patients with rib fractures and accompanying PNX often present with surgical emphysema. The presence of surgical emphysema presents unique challenges when performing an ultrasound-guided paravertebral block. First, surgical emphysema interferes with the ultrasound interpretation of a pneumothorax. Second, strong reflections or echoes show on the ultrasound image as white and weaker reflections as gray. A soft tissue air boundary reflects 99% of the beam, thus none is available to reflect back to the probe [11].

All the above factors degrade the ultrasound image, leading to poor visualization of targeted area and inability or difficulty in performing an ultrasound-guided procedure. Saranteas et al. presented two case reports where the presence of subcutaneous air and surgical emphysema resulted in difficulty in the visualization of the structures below [12]. In the medical literature, authors have reported difficulty in obtaining central line access due to the presence of air in the tissues [13].

Unfortunately, there is not much that can be done if the air obscures the image, other than scanning cephalad to caudad to identify areas where the visualization is better. The operator can then utilize these specific windows to image the space. Alternately, using a landmark approach to place the block may be the only option. 

Obesity

Obesity is a major medical problem and presents unique challenges for a proceduralist. While technology has come a long way over the past years with the development of new machines, many shortcomings still exist.

Ultrasound is an important imaging modality that is limited by user experience and skill, as well as patient body habitus and comorbidity. A higher frequency allows for better resolution while sacrificing penetration. This poses a challenge for the physician using ultrasound to perform a nerve block on an obese patient. To clearly visualize structures deeper to the extra subcutaneous adipose tissue, lower frequencies need to be used which results in poor resolution and, therefore, poor quality of the images. The problem here is evident - poor image quality leads to more difficult regional blocks, which ultimately leads to higher failure rates and increased complications. This problem is amplified in trauma patients where (due to the injury suffered by the patient) the anatomy is altered, leading to even more confusion by the practitioner on what the image is showing. 

Obesity increases the acoustic path length, requiring a greater depth of penetration to view a target. Obesity may also have an indirect effect on the underlying muscle thickness and tone [14].

Imaging is degraded in obese individuals due to the following phenomena:

1) Sound attenuation: The ultrasound beam is weakened as it travels through the adipose tissue. The higher the frequency of the probe, the more attenuation is seen [15].

2) Phase aberration of the sound field reduces the quality of the ultrasound because of the uneven speed of sound in the irregular layers of adipose [16].

The following tips provide a better resolution while using ultrasound [17]:

1) Using a low-frequency transducer counteracts sound attenuation,

2) Compressing the tissues with the transducer brings the target in better focus, 

3) Using a large beamwidth of the ultrasound signal,

4) Utilizing advanced technology, like tissue harmonic imaging, aiding in better tissue penetration [18].

Transverse process fractures (TVPFX)

Thoracolumbar spine TVPFXs are markers of major torso trauma and indicate a high-energy mechanism. They may have pulmonary or gastrointestinal dysfunction related to their injuries [19].

Even though they are considered not as painful as rib fractures, they can still cause significant pain requiring careful pain management. TVPFXs can disturb the integrity of the paravertebral space. A TVPFX appears as a gap in the cortex of the rib and may be associated with a localized hematoma, effusion, or soft tissue swelling. Ultrasonography can visualize the costal cartilage, as well as the osseous part of the rib [20].

A paravertebral block (PVB) at the spinous level of a fractured transverse process can be unreliable and difficult due to the presence of bony fragments and/or hematoma. However, in our experience, these patients benefit tremendously from the nerve blocks, even if a successful PVB is not achieved. This may be due to the spread of local anesthetic directly at the site of trauma. 

Whenever feasible, continuous paravertebral catheters should be placed instead of a single injection since the pain from rib fractures affects the patient acutely for several days. Patients aged 65 years or older with two or more rib fractures, those with preexisting pulmonary disease, and patients with evidence of an increased effort of breathing/splinting are good candidates for paravertebral catheters [21]. Multimodal pain management, including non-steroidal anti-inflammatory drugs (NSAIDs), acetaminophen, and short-acting opioids, should be strongly considered.

## Conclusions

In conclusion, US-guided TPVB is a useful and safe technique of providing adequate pain relief in rib fracture patients. These patients have significant pain management needs and require a specialized effort in mobilization, reasonably extending their hospital stay. Since there is a lack of considerable data for ultrasound-guided paravertebral techniques, no studies or review articles have elaborated on the use of ultrasound-guided paravertebral block in rib fracture patients. We hope that this article is able to shed more light on this matter and enable readers to make an educated decision when faced with these challenges.
